# Comparison of SARS-CoV-2- and HCoV-Specific T Cell Response Using IFN-γ ELISpot

**DOI:** 10.3390/diagnostics11081439

**Published:** 2021-08-10

**Authors:** Laura Thümmler, Sina Schwarzkopf, Dietmar Knop, J. Alexander Ross, Victoria Berg, Peter A. Horn, Monika Lindemann

**Affiliations:** 1 Institute for Transfusion Medicine, University Hospital Essen, University of Duisburg-Essen, 45147 Essen, Germany; Laura.Thuemmler@uk-essen.de (L.T.); sina.schwarzkopf@stud-mail.uni-wuerzburg.de (S.S.); Dietmar.Knop@uk-essen.de (D.K.); Peter.Horn@uk-essen.de (P.A.H.); 2 Institute of Cell Biology (Cancer Research), University Hospital Essen, University of Duisburg-Essen, 45147 Essen, Germany; alexander.ross@uni-due.de (J.A.R.); victoria.berg@stud.uni-due.de (V.B.)

**Keywords:** cross-reactivity, ELISpot, human endemic coronavirus

## Abstract

Herd immunity is essential to control severe acute respiratory syndrome coronavirus type 2 (SARS-CoV-2), especially in immunocompromised patients. Convalescent individuals should be vaccinated later due to vaccine shortage, as studies show that neutralizing antibodies generated during infection are stable for at least 6 months. Cellular immunity is also detectable for months. However, there is evidence of cross-reactivity of T cells with human endemic coronaviruses (HCoVs). Here, we show that cross-reactivity—which may prevent the specific detection of SARS-CoV-2-specific T cell responses—can be avoided if cells are stimulated with the N-terminus of the spike protein in IFN-γ ELISpot. In contrast to previous studies, we examined T-cell responses against all four known HCoVs using IFN-γ ELISpot in 19 convalescent volunteers and 10 fully vaccinated volunteers. In addition, we performed Spearman analyses to detect cross-reactivity of T cells. We observed no correlation between T-cell responses against SARS-CoV-2 and human endemic coronaviruses, either in the whole cohort or in the individual groups. The use of the respective stimuli could lead to a more accurate assessment of cellular immunity in recovered individuals. This testing procedure could help to define the best time point at which convalescents should receive SARS-CoV-2 vaccination.

## 1. Introduction

Since the start of the coronavirus disease 2019 (COVID-19) pandemic, more than 3.6 million people have died due to SARS-CoV-2 infection (June 2021). To prevent the spread of infection, state governments have imposed various restrictions [1]. However, only herd immunity is supposed to be capable of ending the pandemic [2,3,4]. Furthermore, it can protect vulnerable groups, such as transplant recipients or other immunocompromised patients, from being infected. Herd immunity exists when a large proportion of the population is immune to a pathogen. This immunity can result from resolved SARS-CoV-2 infection or vaccination [5].

Because there is still a shortage of SARS-CoV-2 vaccines, vaccination needs to be prioritized [6,7]. For this reason, people should not be vaccinated initially after COVID-19 infection, as sufficient immunity is assumed.

There is evidence that immunity to SARS-CoV-2 varies depending on the severity of infection [8]. There are also indications of cross-reactive T cells and antibodies following infection with common cold coronaviruses such as human Coronavirus HCoV-HKU1, HCoV-OC43, HCoV-NL63, and HCoV-229E and with SARS-CoV-2 [5,9,10,11,12,13,14]. HCoV-HKU1 and HCoV-OC43 are closely related to SARS-CoV-2, as all three are betacoronaviruses [15]. Nevertheless, the studies also gave evidence of cross-reactivity with the alphacoronaviruses HCoV-NL63 and 229E. However, for a confident assessment of cellular immunity to SARS-CoV-2, it is of particular importance to use specific assay procedures.

In the current study, we analyzed cellular immunity to SARS-CoV-2 and HCoV in 29 individuals, 19 convalescent, potential plasma donors and 10 vaccinated individuals. COVID-19 infection in the convalescent individuals occurred at a median of 376 days prior to sampling. In the vaccinated group, vaccination dated back a median of 33 days. Both groups were tested by SARS-COV-2- and HCoV-specific interferon (IFN)-γ ELISpot assay to detect possible cross-reactivity of T cells.

## 2. Materials and Methods

### 2.1. Volunteers

As a first group, we included 19 potential plasma donors who recovered from SARS-CoV-2 infection in 2020. The acquisition of volunteers has already been described by Schwarzkopf et al. (2020) [15]. The group contained 12 males and 7 females; the median age of the donors was 51 years (range 28–66). The COVID-19 infection occurred at a median of 376 days (range 291–394) prior to sampling.

As a second group, we included 10 vaccinated volunteers without symptoms of SARS-CoV-2 infection before vaccination. The group was composed of 6 males and 4 females, the median age was 30 years (range 28–60). Cellular immunity of the vaccinated volunteers was analyzed at a median of 33 days (range 27–54) after the second vaccination. Five of them were vaccinated with Comirnaty (BNT162b2, Biontech/Pfizer, Mainz, Germany) and five with the Moderna COVID-19 vaccine (mRNA-1273, Moderna Biotech, Madrid, Spain).

In total, 29 volunteers were tested for cellular immunity against SARS-CoV-2 and HCoV by IFN-γ ELISpot.

The study was approved by the local ethics committee of the University Hospital Essen, Germany (20-9225-BO) and all volunteers provided informed consent to participate in the study. It was performed in accordance with the ethical standards noted in the 1964 Declaration of Helsinki and its later amendments or comparable ethical standards.

### 2.2. ELISpot Assay

**Figure 1:**
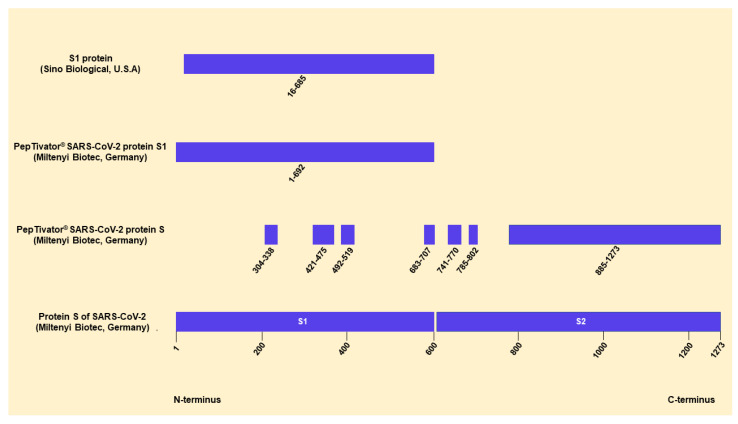
Sequence information of the S antigens of SARS-CoV-2. This figure contains all available information on the S antigens of SARS-CoV-2 used for the ELISpot assays. We modified an image by Miltenyi Biotec (https://www.miltenyibiotec.com/DE-en/products/peptivator-sars-cov-2-prot-s-107090.html#130-126-700, accessed on 29 June 2021), containing information on their PepTivator product and the protein sequence of SARS-CoV.2. The peptide mix (PepTivator) of the S protein (S1/S2) consists mainly of 15-mer sequences with 11 amino acids overlap, covering the immunodominant sequence domains of the surface glycoprotein of SARS-CoV-2. The peptide mix (PepTivator) of the S1 protein (S1) covers the complete N-terminus of the S antigen. The S1 Sino protein (http://www.sinobiological.com, accessed on 27 June 2021) is a recombinant protein expressed in (human) HEK293 cells.

ELISpot stripes containing polyvinylidene difluoride (PVDF) membranes (MilliporeSigma™ MultiScreen™ HTS, Fisher Scientific, Schwerte, Germany) were activated with 50 µL of 35% ethanol for 10 s and washed with distilled water. Plates were then coated for three hours with 60 µL of monoclonal antibodies against IFN-γ (10 µg/mL of clone 1-D1K, Mabtech, Nacka, Sweden). Thereafter, ELISpot plates were washed and then blocked with 150 µL AIM-V^®^ (Thermo Fisher Scientific, Grand Island, NY, USA). After 30 min at 37 °C, AIM-V^®^ was discarded and duplicates of 250,000 peripheral blood mononuclear cells (PBMC) were grown in the presence or absence of either PepTivator^®^ SARS-CoV-2 protein S1/S2 or protein S1 (600 pmol/mL of each peptide, Miltenyi Biotec, Bergisch Gladbach, Germany) in 150 µL of AIM-V^®^. Single cell cultures of 250,000 PBMC were grown in the presence or absence of either an S1 protein (called S1 Sino from here on, 4 µg/mL, Sino Biological, Wayne, PA, USA) and an S1 protein of HCoV-HKU1, HCoV-OC43, HCoV-NL63 and HCoV-229E (4 µg/mL of each peptide, Sino Biological, Beijing, China) in 150 µL of AIM-V^®^. The peptide mix (PepTivator^®^) of the S1/S2 protein covers the immunodominant domains and the C-terminus. The peptide mix of the S1 protein and the S1 protein from Sino Biological both cover the N-terminus. The S1 Sino protein is a recombinant protein expressed in (human) HEK293 cells. The S1 protein of each tested HCoV spans the N-terminus (Figure 1). After 19 h of incubation at 37 °C, the ELISpot plates were washed and captured IFN-γ was detected by incubation for one hour with 50 µL of the alkaline phosphatase-conjugated monoclonal antibody against IFN-γ (clone 7-B6-1, Mabtech, Stockholm, Sweden), diluted 1:200 with PBS plus 0.5% bovine serum albumin (BSA). After further washing, 50 µL of nitro blue tetrazolium/5-bromo-4-chloro-3-indolyl-phosphate (NBT/BCIP) was added and purple spots appeared within 7 min. Spot numbers were analyzed by an ELISpot reader (AID Fluorospot, Autoimmun Diagnostika GmbH, Strassberg, Germany). Mean values of duplicate cell cultures were considered. SARS-CoV-2- and HCoV-specific spots were determined as stimulated minus non-stimulated values (spots increment). The cut-off definition was based on negative control values (non-stimulated cultures) and on the consideration that threefold higher values for stimulated vs. non-stimulated cells are frequently considered as a positive response in cellular assays. The negative controls had on average 0.50 spots (range 0–12) and its 3-fold standard deviation was 3 × 1.34 spots = 4.02 spots (which we considered as background of the negative controls). As we used increment values, a threefold higher value vs. background means 3 × 4.02 spots minus 1 × 4.02 spots, i.e., 8.04 spots increment. We therefore chose 8 as the cut-off for positivity, together with the criterion of threefold higher values of stimulated vs. non-stimulated cultures [16].

### 2.3. Alignments

The open source software Jalview (http://www.jalview.org, accessed on 27 June 2021) [17] was used for the alignments. Sequences were obtained from National Center for Biotechnology Information (NCBI, Appendix A).

### 2.4. Statistical Analysis

Statistical analysis was performed using GraphPad Prism 8.0.1 (San Diego, CA, USA) software. We used Spearman correlation and linear regression analysis for numerical variables. The analysis of categorical variables was performed by Mann–Whitney test or 1-way ANOVA (Friedman test) with Dunn’s correction for multiple comparisons, as appropriate. Two-sided *p* values < 0.05 were considered significant.

## 3. Results

**Figure 2:**
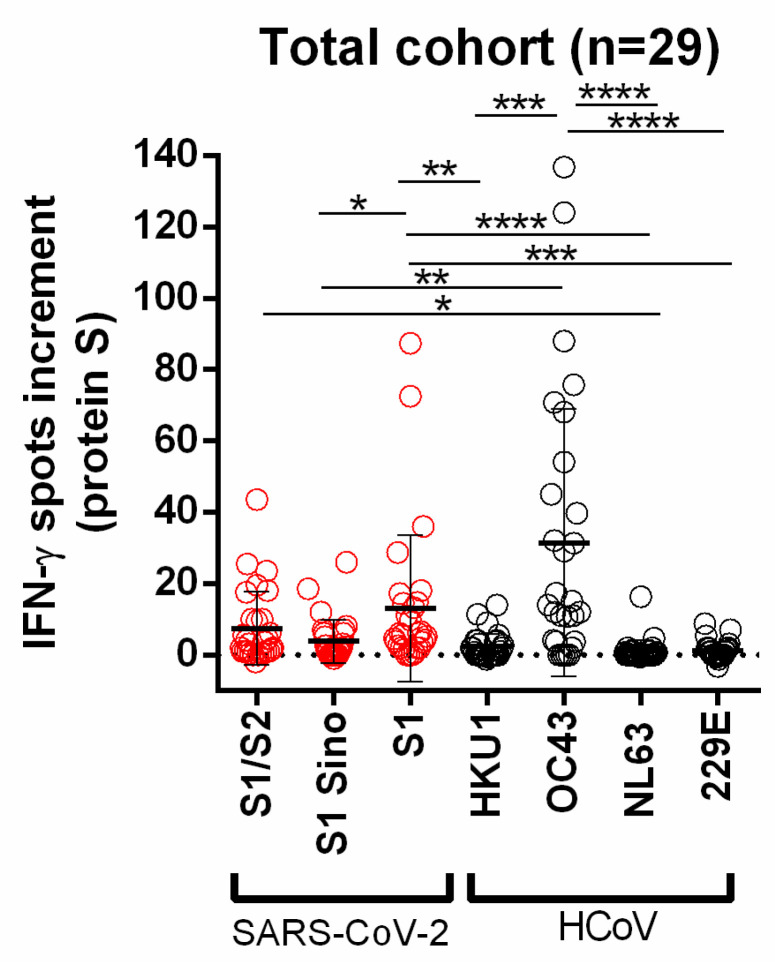
Distribution of SARS-CoV-2- and HCoV-specific ELISpot responses in all volunteers. Red circles indicate IFN-γ spots increment upon stimulation with S1/S2 peptide mix, S1 Sino, and S1 peptide mix of SARS-CoV-2. Gray circles indicate IFN-γ spots increment upon stimulation with S1 proteins of HCoV-HKU1, HCoV-OC43, HCoV-NL63, and HCoV-229E. Responses were compared by 1-way ANOVA (Friedman test) with Dunn’s correction (* *p* < 0.05, ** *p* < 0.01, *** *p* < 0.001, **** *p* < 0.0001). Horizontal lines indicate mean values, error bars indicate the standard deviation. The dotted line represents the zero line.

In the present study, 19 potential convalescent plasma donors and 10 vaccinated individuals were tested for cellular immunity to SARS-CoV-2 and different HCoVs to observe or exclude possible T-cell cross-reactivity. For SARS-CoV-2, the S1 peptide mix induced the highest ELISpot response (mean = 13.1 spots increment) and for the human endemic coronaviruses the S1 protein of HCoV-OC43 (mean = 31.4 spots increment). Spot numbers after stimulation with the S1 peptide mix of SARS-CoV-2 vs. S1 Sino were significantly higher (*p* < 0.05) and spot numbers after stimulation with HCoV-OC43 vs. HCoV-HKU1, HCoV-NL63 and HCoV-229E (*p* < 0.001) (Figure 2).

**Figure 3:**
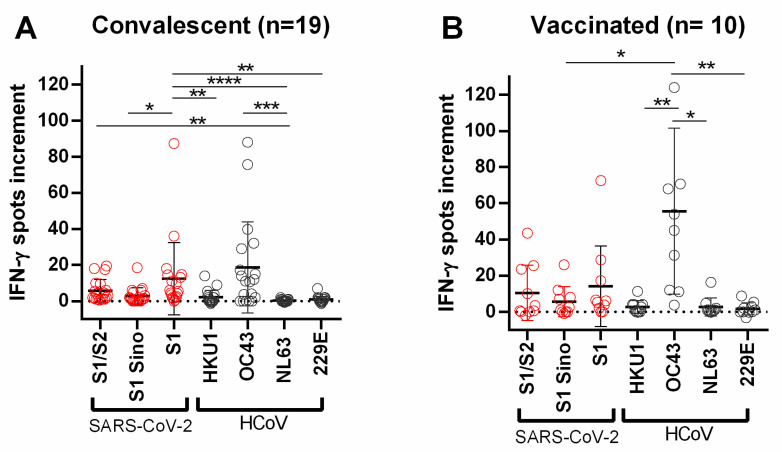
Distribution of SARS-CoV-2- and HCoV-specific ELISpot responses in (**A**) convalescent and (**B**) vaccinated volunteers. Red circles indicate IFN-γ spots increment upon stimulation with S1/S2 peptide mix, S1 Sino, and S1 peptide mix of SARS-CoV-2. Gray circles indicate IFN-*γ* spots increment upon stimulation with S1 proteins of HCoV-HKU1, HCoV-OC43, HCoV-NL63, and HCoV-229E. Responses were compared by 1-way ANOVA (Friedman test) with Dunn’s correction (* *p* < 0.05, ** *p* < 0.01, *** *p* < 0.001, **** *p* < 0.0001). Horizontal lines indicate mean values, error bars indicate the standard deviation. The dotted line represents the zero line. The dotted line represents the zero line.

Using the cut-off of eight spots increment, stimulation with the S1 protein of HCoV-OC43 showed the highest percentage of positive responses with 68.9% (HCoV-HKU1: 6.9%; HCoV-NL63: 3.5%; HCoV-229E: 3.4%). Stimulation with S proteins/peptide mixes of SARS-CoV-2 showed the highest percentage with the S1 peptide mix at 37.9% (S1/S2: 31.0%; S1 Sino: 10.3%) (Table S1). We also analyzed the two groups, potential convalescent plasma donors and vaccinated volunteers, separately. The maximum number of spots increment was observed after stimulation with the S1 peptide mix of SARS-CoV-2 (convalescent plasma donors: mean = 12.5; vaccinated volunteers: mean = 14.2) and the S1 protein of HCoV-OC43 (convalescent plasma donors: 18.6; vaccinated volunteers: mean = 55.6). In the group of convalescent plasma donors, spot numbers after stimulation with the S1 peptide mix of SARS-CoV-2 vs. S1 Sino were significantly higher (*p* < 0.05) than spot numbers after stimulation with HCoV-OC43 vs. HCoV-NL63 (*p* < 0.001). Moreover, in vaccinated volunteers, differences between responses towards HCoV-OC43 and HCoV-HKU1, HCoV-NL63 and HCoV-229E reached statistical significance (*p* < 0.05) (Figure 3).

**Figure 4:**
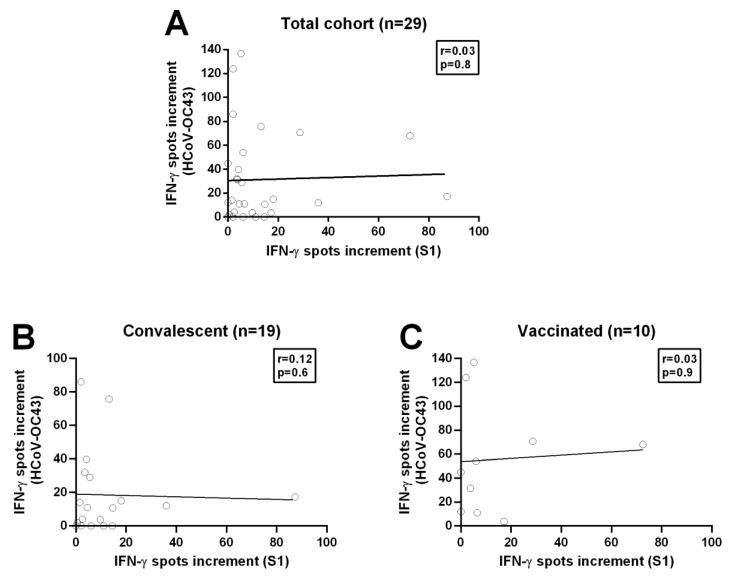
Spearman correlation analysis on T cell immunity towards SARS-CoV-2 and HCoV-OC43. (**A**) Spearman correlation between T cell immunity against the S1/S2 peptide mix of SARS-CoV-2 and the N-terminus of the S1 protein of HCoV-OC43 in the total cohort (*n* = 29). (**B**) Spearman correlation between T cell immunity against the S1/S2 peptide mix of SARS-CoV-2 and the N-terminus of the S protein of HCoV-OC43 in potential convalescent plasma donors (*n* = 19). Please note that the scale is different. (**C**) Spearman correlation between T cell immunity against the S1/S2 peptide mix of SARS-CoV-2 and the N-terminus from the S protein of HCoV-OC43 in completely vaccinated volunteers (*n* = 10). The figure is representative of all Spearman correlation analyses performed. The black line indicates the regression line.

**Figure 5:**
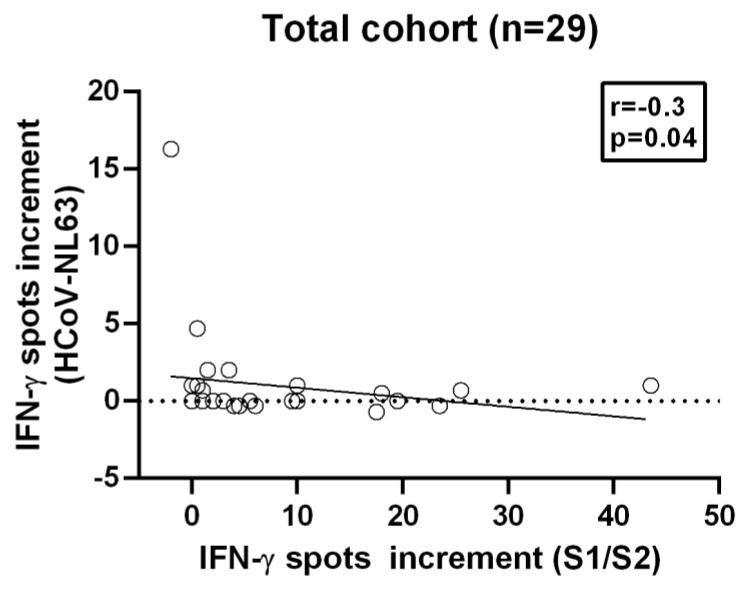
Spearman correlation analysis on T cell immunity towards SARS-CoV-2 and HCoV-NL63. Spearman correlation between T cell immunity against the S1/S2 peptide of SARS-CoV-2 and the N-terminus of the S1 protein of HCoV-NL63 in all volunteers. The dotted line represents the zero line. The black line indicates the regression line.

We started with Spearman correlation using these two strongest stimuli and analyzed the total cohort and additionally the separate groups, recovered and vaccinated volunteers. However, there was no correlation between the spots increment upon stimulation with SARS-CoV-2-specific S1 peptide mix and stimulation with the S1 protein of HCoV-OC43, neither for the analysis of the total cohort nor for the separate analysis of recovered and vaccinated volunteers (total cohort: correlation coefficient r = −0.18, *p*-value = 0.3; convalescent plasma donors: correlation coefficient r = −0.12, *p*-value = 0.6; vaccinated volunteers: correlation coefficient r = −0.05, *p*-value = 0.9;) (Figure 4, Table S2–S4). We furthermore analyzed all tested peptide mixes/proteins by Spearman analysis. In the total cohort, we found only a negative correlation (r = −0.4, *p*-value = 0.04) (Figure 5) between the S1/S2 peptide mix of SARS-CoV-2 and the S1 protein of HCoV-NL63; no other correlations were detected. The Spearman analysis for the separate groups showed no correlation.

**Figure 6:**
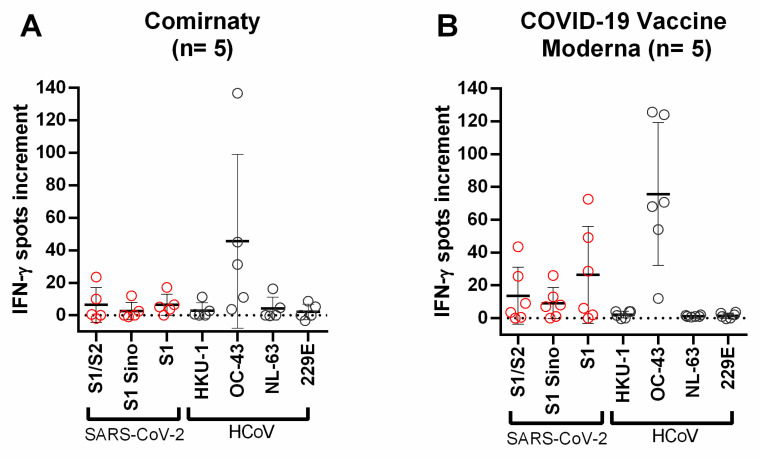
Distribution of SARS-CoV-2- and HCoV-specific ELISpot responses in vaccinated volunteers. (**A**) Distribution of ELISpot responses upon stimulation with different S proteins in volunteers vaccinated with Comirnaty (*n* = 5). (**B**) Distribution of ELISpot responses upon stimulation with different S proteins in volunteers vaccinated with Moderna COVID-19 vaccine (*n* = 5). Red circles indicate IFN-*γ* spots increment upon stimulation with S1/S2 peptide mix, S1 Sino, and S1 peptide mix of SARS-CoV-2. Gray circles indicate IFN-*γ* spots increment upon stimulation with S1 proteins of HCoV-HKU1, HCoV-OC43, HCoV-NL63, and HCoV-229E. Responses in the three groups of volunteers were compared by Mann–Whitney test. Horizontal lines indicate mean values, error bars indicate the standard deviation. The dotted line represents the zero line. The black line indicates the regression line.

Since the group of vaccinated individuals could be divided into two groups, depending on the vaccine, we analyzed whether there was a difference in cross-reactivity. The Spearman correlation showed no significant correlation in either group between the stimulation of the sample with SARS-CoV-2-specific S protein/peptide mixes and stimulation with S1 proteins of HCoVs. However, it was noticeable that volunteers vaccinated with the Moderna COVID-19 vaccine had higher spots increment for the S proteins of SARS-CoV-2 compared to those vaccinated with Comirnaty (Figure 6). The volunteers receiving the Moderna vaccine showed a mean number of spots increment for S1/S2 peptide mix of 14.6, for S1 Sino of 8.4 and for S1 peptide mix of 21.8, while those vaccinated with Comirnaty showed a mean number for S1/S2 peptide mix of 6.4, for S1 Sino of 2.7, and for S1 peptide mix of 6.5. This difference did not reach statistical significance, presumably because the number of volunteers was too low.

**Figure 7:**
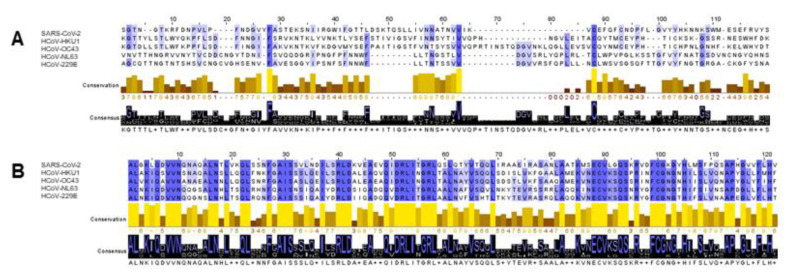
Alignment of amino acid sequences of the S proteins of SARS-CoV-2, HCoV-HKU1, HCoV-OC43, HCoV-NL63, and HCoV-229E. (**A**) Representative section of 120 amino acids of the alignment in the region of the N-terminus. (**B**) Representative section of 120 amino acids of the alignment in the region of the C-terminus. The alignments were generated with the open source software Jalview. The matching amino acids are highlighted in blue. The darker the shade of blue, the higher the percentage of the match. High conservation is represented by a yellow bar, lower conservations by brown bars. The consensus shows the predominant amino acids.

In addition, we compared the amino acid sequences of the S protein of SARS-CoV-2 with the amino acid sequences of the S proteins of HCoVs using Mafft alignment with the software Jalview. The results show strong conservation of the sequences in the C-terminus, but not in the N-terminus, which is contained in the S1 peptide mix, the S1 Sino and the S1 proteins of HCoVs, which we used for stimulation (Figure 7).

Overall, there was no positive correlation—indicating cross-reactivity—between SARS-CoV-2-specific and HCoV-specific T cells in our established IFN-**γ** ELISpot. This indicates that stimulation with proteins from the N-terminus of the S1 protein allows specific results and thus an accurate assessment of cellular immunity after SARS-CoV-2 infection or vaccination.

## 4. Discussion

In this study, we tested 19 potential convalescent plasma donors and 10 fully vaccinated volunteers for cellular immunity against SARS-CoV-2 and various HCoVs using IFN-γ ELISpot.

Since the infection in the potential convalescent plasma donors already occurred approximately one year ago, specific responses of the memory T cells can be assumed here. Specific memory T cells have already been found in SARS-CoV-1 infected patients, which were still detectable 10 years after infection [18,19,20]. Due to the similarity of SARS-CoV-1 and SARS-CoV-2, this could indicate a long-lasting immune response. In vaccinated individuals, the examination of cellular immunity after one year is advisable to provide insights into the duration of immunity to SARS-CoV-2 by the novel mRNA vaccines. However, in both groups, protection against reinfection due to T cells needs to be further explored.

The vaccinated donors were tested four to six weeks after their second vaccination, as this is when the peak adaptive immune response occurs. The convalescent individuals were tested approximately one year after infection. These temporal factors limit comparisons within and between groups, but provide insight into the duration of cellular responses after infection. Based on these data, a comparison of cellular immunity at different time points is possible, allowing an estimate of the best time to vaccinate convalescent individuals. Currently, an interval of one year from infection is considered to be a good time for vaccination.

In both the overall cohort and the separate groups, the number of spots increment for HCoV-OC43 was comparatively high. Additionally, when using the cut-off we defined, stimulation with the S1 protein of HCoV-OC43 showed the highest percentage with 68.9% positive results. These results clearly indicate that our cohort had predominantly been infected by HCoV-OC43. These results are also consistent with the findings of the Clinical Virology Network from Germany, which are investigating the seasonal activity of HCoVs in Germany and a study from Kissler et al. in the USA [14]. In this case, too, Germany had the highest number of infections with HCoV-OC43 [21].

Previous studies have examined antibody titers to endemic HCoVs and T cell responses to alphacoronaviruses but not to betacoronaviruses [5,9,10]. Spearman analysis revealed a negative correlation between the S1/S2 peptide mix and the S1 protein of the betacoronavirus HCoV-NL63. However, since this correlation only occurs in the Spear-man analysis in the total cohort and shows a weak correlation coefficient of r = −0.3 with a *p*-value = 0.04, the result is most likely of no significance, especially when considering multiple testing. In previous studies on the cross-reactivity of SARS-CoV, which exhibits strong conservation to SARS-CoV-2, Vlasova et al. found no cross-reactivity between SARS-CoV and HCoV-NL63 mediated by the S protein [22]. Since both the N- and C-terminus of the protein are tested in the S1/S2 peptide mix, cross-reactivity of the T cells could occur here. In further experiments, the N- and C-terminus of the S protein of SARS-CoV-2 could be used separately for T cell stimulation. However, because we used commercial antigens, this possibility remained beyond our reach.

Individuals vaccinated with the Moderna COVID-19 vaccine showed overall higher spot numbers for the tested S proteins and peptides of the S protein of SARS-CoV-2 compared with individuals vaccinated with Comirnaty. This could be due to the different amount of mRNA administered during vaccination. In the case of Comirnaty, 30 µg mRNA/dose is applied, whereas in the case of the Moderna COVID-19 vaccine, 100 µg mRNA/dose is administered [23,24]. Another reason could be the different modulation of the mRNA. Of note, both vaccinated groups had a comparable age and sex distribution. The distance to the second vaccination did not differ significantly between the two groups (Comirnaty: 40 days, Moderna COVID-19 vaccine Moderna: 32 days). A decrease in cellular immunity within one week is considered unlikely. However, further studies with more subjects are necessary, as well as a comparison between vaccines receiving mRNA and vector-based vaccines. The high spot numbers after stimulation with HCoV-OC43 appear as independent from vaccination, as they were present in all individuals tested. This is most likely due to the high rate of HCoV-OC43 infection [21].

Previous studies postulated T-cell cross-reactivity [5,9,10,11], which could not be demonstrated in the present study. This could be caused by the fact that we stimulate the cells in our test system with the N-terminus of the respective S protein. Braun et al. also found less cross-reactive T cells when stimulated with the N-terminus of the respective S protein [9]. This is mainly due to the fact that the N-termini of the S proteins are barely conserved, whereas the C-termini show strong conservation between the individual HCoVs as well as between the HCoVs and the SARS-CoV-2 S protein.

Nevertheless, cross-reactivity of T cells can be helpful both in protecting against reinfection with COVID-19 and in fighting infection. However, cross-reactivity may also lead to an exaggerated immune response, which would significantly worsen the course [10]. To date, it has not been conclusively determined whether cross-reactivity of T cells moderates or exacerbates the course of COVID-19.

In conclusion, our IFN-γ ELISpot using the SARS-CoV-2 S1 peptide mix appeared as specific, showing no detectable correlation with any of the human endemic coronaviruses. It may thus be suitable to determine cellular immunity after SARS-CoV-2 infection or vaccination.

## Data Availability

The data can be obtained from M.L. upon request.

## Supplementary Materials

The following are available online at https://www.mdpi.com/article/10.3390/diagnostics11081439/s1, Table S1: Spearman analysis of the total cohort; Table S2: Spearman analysis of the convalescent volunteers; Table S3: Spearman analysis of the vaccinated volunteers; Table S4: Distribution of positive T cell responses in the total cohort.

## Appendix A

Links to NCBI:

SARS-CoV-2: https://www.ncbi.nlm.nih.gov/nuccore/MN908947, accessed on 26 June 2021.

HCoV-HKU1: https://www.ncbi.nlm.nih.gov/protein/YP_173238.1, accessed on 26 June 2021

HCoV-OC43: https://www.ncbi.nlm.nih.gov/protein/AVR40344.1, accessed on 26 June 2021.

HCoV-NL63: https://www.ncbi.nlm.nih.gov/protein/APF29071.1, accessed on 26 June 2021.

HCoV-229E: https://www.ncbi.nlm.nih.gov/protein/APT69883.1, accessed on 26 June 2021.
