# Supplementary material for: Comparison of SARS-CoV-2- and HCoV-Specific T Cell Response Using IFN-γ ELISpot
Source: Diagnostics (Basel). 2021 Aug 10;11(8):1439. doi: 10.3390/diagnostics11081439 (PMC8394367; doi:10.3390/diagnostics11081439)
Supplement: Supplementary file 1 [file diagnostics-11-01439-s001.zip › diagnostics-1304805-supplementary.pdf]

Table S1: Distribution of positive T cell responses in IFN-γ ELISpot. The percentage of results considered positive according to the cut-off definition was calculated. (Cut-off definition: The negative controls had on average 0.50 spots (range 0-12) and its 3-fold standard deviation was 3 x 1.34 spots = 4.02 spots (which we considered as background of the negative controls). As we used increment values, a threefold higher value vs. background means 3 x 4.02 spots minus 1 x 4.02 spots, i.e., 8.04 spots increment.)

Coronavirus	Percentage of positive responses
S1/S2 peptide mix	31.0%
S1 peptide mix	37.9%
S1 Sino protein	10.3%
HCoV-HKU1	6.9%
HCoV-OC43	68.9%
HCoV-NL63	3.5%
HCoV-229E	3.4%

Table S2: Spearman analysis of the total cohort. In each case, spots increment upon stimulation with SARS-CoV-2 proteins/peptide mixtures was tested against spots increment upon stimulation with the S protein of HCoVs.

SARS-CoV-2 vs. HCoV	Correlation coefficient r	p-value
S1/S2 vs. HKU1	-0.2656	0.1638
S1 vs. HKU1	-0.1935	0.3145
S1 Sino vs. HKU1	-0.0740	0.7028
S1/S2 vs. OC43	-0.1802	0.3496
S1 vs. OC43	0.0319	0.8695
S1 Sino vs. OC43	0.1410	0.4658
S1/S2 vs. NL63	-0.3686	0.0491
S1 vs. NL63	-0.2345	0.2208
S1 Sino vs. NL63	0.0812	0.6754
S1/S2 vs. 229E	0.0234	0.9040
S1 vs. 229E	-0.0811	0.6760
S1 Sino vs. 229E	0.1200	0.5351

Table S3: Spearman analysis of the convalescent volunteers. In each case, spots increment upon stimulation with SARS-CoV-2 proteins/peptide mixtures was tested against spots increment upon stimulation with the S protein of HCoVs.

SARS-CoV-2 vs. HCoV	Correlation coefficient r	p-value
S1/S2 vs. HKU1	-0.2591	0.2840
S1 vs. HKU1	-0.2756	0.2534
S1 Sino vs. HKU1	-0.3076	0.2001
S1/S2 vs. OC43	-0.1224	0.6177

S1 vs. OC43	0.1204	0.6234
S1 Sino vs. OC43	0.1684	0.4908
S1/S2 vs. NL63	-0.2928	0.2239
S1 vs. NL63	-0.3182	0.1842
S1 Sino vs. NL63	-0.0196	0.9367
S1/S2 vs. 229E	-0.1838	0.4513
S1 vs. 229E	-0.2674	0.2683
S1 Sino vs. 229E	-0.1379	0.5734

Table S4: Spearman analysis of the vaccinated volunteers. In each case, spots increment upon stimulation with SARS-CoV-2 proteins/peptide mixtures was tested against spots increment upon stimulation with the S protein of HCoVs.

SARS-CoV-2 vs. HCoV	Correlation coefficient r	p-value
S1/S2 vs. HKU1	-0.3313	0.3484
S1 vs. HKU1	-0.0187	0.9616
S1 Sino vs. HKU1	0.3963	0.2603
S1/S2 vs. OC43	-0.0609	0.8706
S1 vs. OC43	0.3040	0.9382
S1 Sino vs. OC43	0.0184	0.9730
S1/S2 vs. NL63	-0.4242	0.2204
S1 vs. NL63	-0.2130	0.5484
S1 Sino vs. NL63	0.0935	0.8008
S1/S2 vs. 229E	0.0741	0.8427
S1 vs. 229E	0.1662	0.6456
S1 Sino vs. 229E	0.5031	0.1434
